# Number of Lawyers, Ministers and Doctors in the United States

**Published:** 1885-09-20

**Authors:** 


### NUMBER OF LAWYERS, MINISTERS, AND DOCTORS IN THE UNITED STATES.

| | |
|---|---|
| Number of Doctors in 1850 | 40,564 |
| Number of Lawyers in 1850 | 23,939 |
| Number of Ministers in 1850 | 26,842 |
| Number of Doctors in 1880 | 85,671 |
| Number of Lawyers in 1880 | 64,137 |
| Number of Ministers in 1880 | 64,698 |

There was in 1880 one doctor to 585 people; one lawyer to 782, and one minister to 775 of the population. Among the numbers thus recorded are 2,432 female doctors; 75 female lawyers, and 165 female ministers. It is believed that, under the prevailing sentiment for Woman's Rights, the proportion of females in the learned professions in 1890 will be largely increased.

